# Case Report: Ultrasound-Guided Percutaneous Microwave Ablation of Focal Nodular Hyperplasia in a 9-Year-Old Girl

**DOI:** 10.3389/fped.2021.710779

**Published:** 2021-07-21

**Authors:** Zhiguang Yao, Qingjing Zeng, Xuan Yu, Shulian Lin, Shuanglan Jiang, Da Ma, Kai Li

**Affiliations:** ^1^The Third Affiliated Hospital of Sun Yat-sen University, Guangzhou, China; ^2^Dongguan Children's Hospital, Dongguan, China

**Keywords:** focal nodular hyperplasia, ultrasonics, ablation, pediatrics, case report

## Abstract

Focal nodular hyperplasia (FNH) is a rare benign tumor-like space-occupying lesion of the liver that is especially rare in children. Since there have been no reports of malignant progression of this disease and these lesions remain unchanged for a long period of time or even disappear in some cases, it remains controversial whether clinical treatment is needed. However, if the diagnosis is unclear, the patient has symptoms or the lesion becomes enlarged during follow-up, clinical treatment should be considered. Here, we report the first case of FNH near the gallbladder treated by ultrasound-guided percutaneous microwave ablation (MWA) in a 9-year-old girl.

## Introduction

Focal nodular hyperplasia (FNH) is a clinically rare benign lesion of the liver that is especially uncommon in children, accounting for approximately 2–7% of pediatric liver tumors (1–5). There are reports of approximately 300 cases of FNH in children. Although the exact etiology and mechanism of FNH remain unclear, the most widely accepted theory is that FNH develops from non-neoplastic hyperplasia of the hepatic parenchyma caused by hepatic vascular malformation or injury (1, 5–7). The main causes include anomalous aortic vascularization, secondary thrombosis, reactive hyperplasia after hepatocellular injury caused by vasculitis, or abnormal blood perfusion, involving either the portal veins or arteries (3). Treatment options for FNH include open surgery, laparoscopic surgery, radiofrequency ablation (RFA), transarterial embolization (TAE), or conservative observation. According to related studies (1, 4, 8–11), the main reasons for clinical treatment are as follows: (i) unclear diagnosis; (ii) obvious symptoms; (iii) tumor larger than 5 cm or causing compression of adjacent organs; and (iv) tumor enlargement during follow-up. Due to its minimal invasiveness and reliable efficacy, percutaneous thermal ablation has gradually become more common for the treatment of FNH in adults, with satisfactory results. Herein, for the first time, we report a case of FNH near the gallbladder treated by ultrasound-guided percutaneous microwave ablation (MWA) in a 9-year-old girl and describe this novel approach for treating FNH in pediatric patients.

## Case Presentation

**Figure 1:**
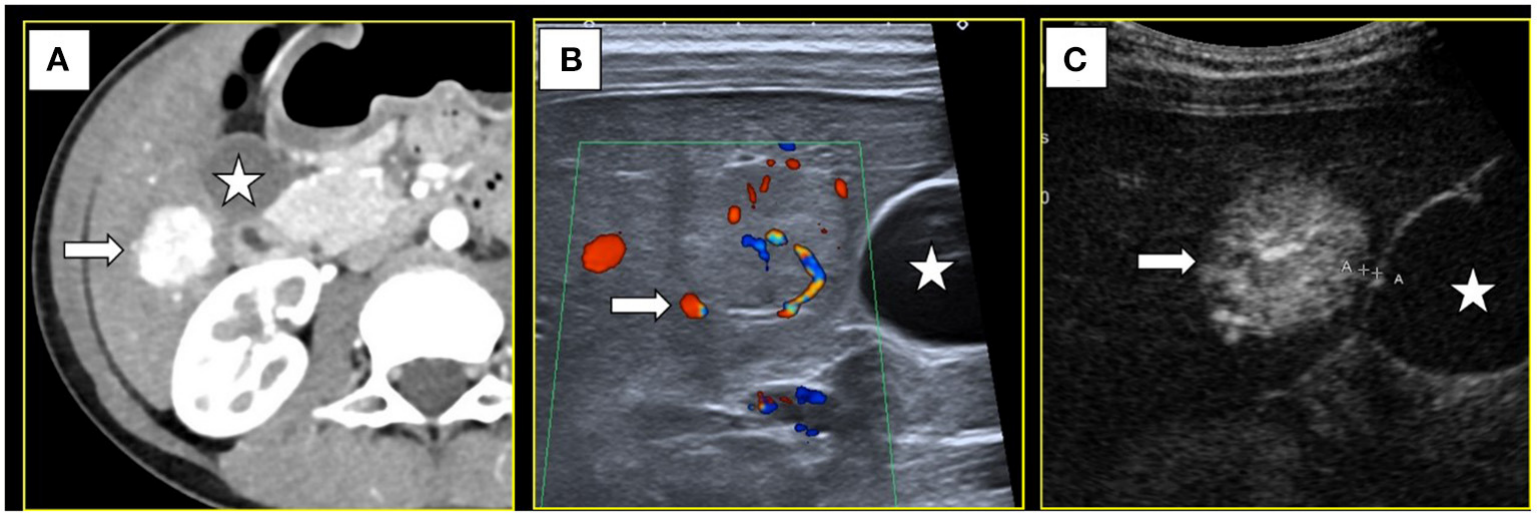
**(A)** Computed tomography showed a S5 lesion (→) with hyper-enhancement in arterial phase and near the gallbladder(⋆). **(B)** Color Doppler ultrasound showed the feeding artery (→). **(C)** Contrast-enhanced ultrasound showed the lesion had hyper-enhancement in arterial phase (→) and only 1mm away from the gallbladder(⋆).

A 9-year-old girl was admitted to Dongguan Children's Hospital 6 months ago due to breast development. Since the patient had no other obvious symptoms, we did some laboratory tests to help diagnose. A gonadotropin releasing hormone (GnRH) stimulation test showed that the peak values of luteinizing hormone (LH) and follicle-stimulating hormone (FSH) were 8.03 and 14.97 IU/L, with an LH/FSH ratio of 0.53. Based on her premature breast development, test results and the absence of relevant personal or family history, she was diagnosed with precocious puberty and treatment with a monthly intramuscular injection of triptorelin (decapeptyl) was initiated. The patient also received imaging examinations to exclude underlying diseases. Unexpectedly, ultrasonography indicated a single, solid, focal liver lesion in S5. Enhanced computed tomography (CT) showed hyperenhancement of the lesion in the arterial phase, which was 17mm × 18mm in size, with a hypodense central stellate scar visible inside the region (Figure 1A). The lesion became isodense in portal venous and delayed phases, and the stellate scar showed slight enhancement. A diagnosis of FNH was considered. CT re-examination 6 months later showed that the lesion had increased to 23mm × 19mm × 18mm in size. According to ultrasound, the lesion was non-homogeneous, slightly hyperechoic with an unclear boundary, and located 1mm away from the gallbladder and 3mm away from the colon. Color Doppler ultrasound revealed spoke-wheel blood flow, with the feeding artery being from the S5 hepatic artery branch (Figure 1B). Contrast-enhanced ultrasound (CEUS) indicated a radiant artery and hyperenhancement of the lesion, which was 29mm × 25mm in size, in the arterial phase (Figure 1C), portal phase and delayed phase.

An APLIO I700 ultrasound system with a PVT-674BT 10C3 abdominal probe (Canon, Japan, 2020), incisive CT (PHILIPS, 2020), and definition flash CT (SOMATOM, 2017) were used for diagnosis. A 100A1 microwave generator (2,450 MHz) and an ECO-100CI10 MWA antenna (Yigao, Nanjing, China) were employed for MWA. SonoVue (Bracco, Italy) was utilized as the contrast agent for ultrasound and was prepared as a suspension with 5 ml of 0.9% normal saline (N.S.). For transvenous use of the contrast agent, 1 ml of SonoVue was injected through a central venous catheter, followed by 5 ml of N.S. For intracavitary use, the ratio of N.S. and SonoVue was 100:1. A coaxial needle (15 G) and an automatic biopsy gun (18 G, BARD, USA) were applied for biopsy and a central venous catheterization set (Arrow, USA) was used for abdominal cavity intubation.

**Figure 2:**
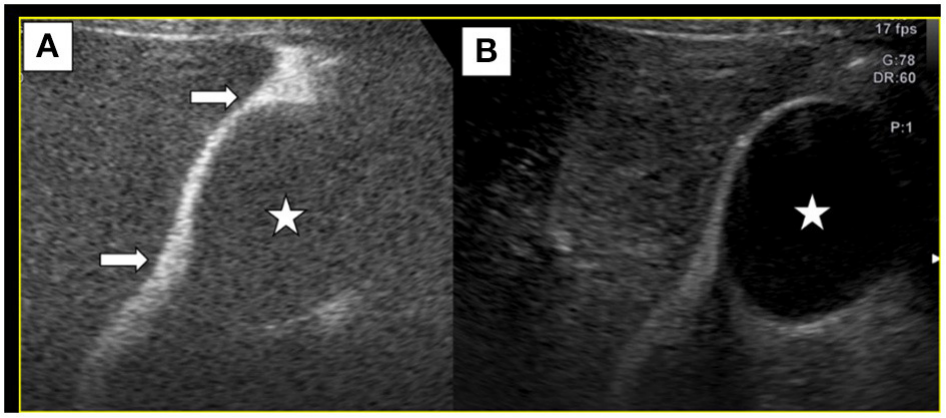
Normal saline and SonoVue (100:1) were infused into abdominal cavity. Contrast-enhanced ultrasound **(A)**, along with B-mode ultrasound **(B)** showed that SonoVue distribution between the gallbladder(⋆) and the liver (→), suggesting that ascites could separate the gallbladder wall away from the liver to avoid thermal injury.

Artificial ascites-assisted ultrasound-guided MWA and biopsy were carried out under general endotracheal anesthesia. Under ultrasound guidance, we performed abdominal cavity intubation, and SonoVue in N.S. at 40°C was infused into the abdominal cavity. Under CEUS, we observed hyperechoic contrast agent distribution between the gallbladder and the hepatic margin close to the lesion, indicating that this fluid could provide a cooling effect during thermal ablation (Figure 2). We also inserted a guide wire near the position of the gallbladder and then inserted an expansion tube, which were used together to push the gallbladder wall away to avoid thermal injury.

**Figure 3:**
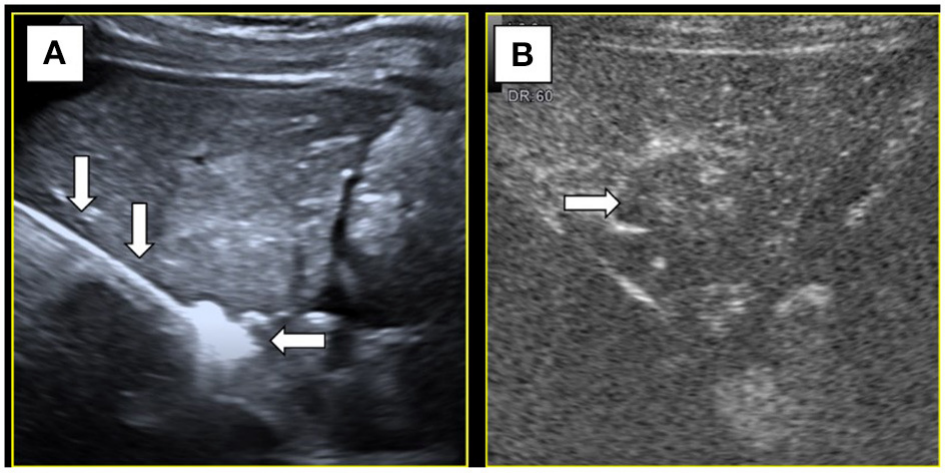
**(A)** Microwave antenna(↓) punctured at the feeding artery and the hyperechoic caused by ablation(←). **(B)** After ablation of the feeding artery, contrast-enhanced ultrasound showed no blood supply (→) inside the tumor.

**Figure 4:**
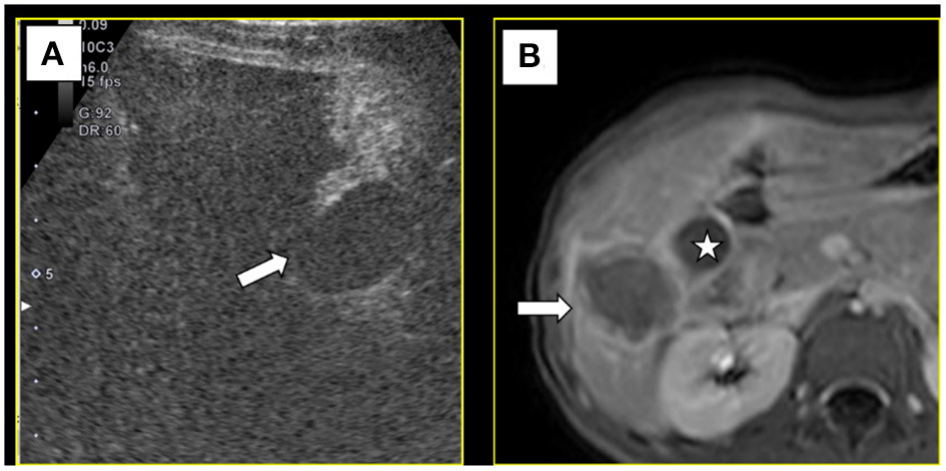
**(A)** After ablation, contrast-enhanced ultrasound showed blood supply in gallbladder wall (→), suggesting the gallbladder wall had no necrosis. **(B)** Magnetic resonance imaging 1 month later showed the lesion was completely ablated (→) and the gallbladder(⋆) was not injured.

Under color Doppler ultrasound guidance, the microwave antenna was inserted directly to the site of the feeding artery (Figure 3A), and after 2 min of ablation at 60 W, CEUS showed no blood flow perfusion in the tumor (Figure 3B). Three biopsy specimens were then collected using a coaxial needle and an automatic biopsy gun. The microwave antenna was inserted through the coaxial needle to ablate the biopsy needle track after ablating the lesion. After biopsy, the tumor border near the gallbladder was ablated, followed by the peripheral part of the tumor and, finally, the interior of the tumor. After ablation, complete ablation of the whole lesion was observed by CEUS. Additionally, the gallbladder wall showed blood perfusion, indicating that the thermal ablation procedure did not cause necrosis in the gallbladder wall (Figure 4A). Moreover, the contrast agent did not enter the ascites, which indicated no needle track bleeding.

Post-operative medications included cefixime granules as an anti-inflammatory agent and compound glycyrrhizin capsules to protect liver function. The abdominal drainage tube was removed on the second day after the operation, and the patient was discharged on the sixth day. Examination by magnetic resonance imaging (MRI) 1 month later showed that the tumor was completely ablated and that the gallbladder was not injured (Figure 4B). Based on ultrasonography after another 3 months, there were no significant changes in the ablation zone and no blood flow signal inside it. Additionally, the gallbladder exhibited no obvious abnormity.

## Discussion

Approximately two-thirds of liver tumors in pediatric patients are malignant, whereas FNH accounts for only 2–7% (1). Thus, the accuracy of diagnosis is of great importance when encountering a hepatic tumor in a child. Here, we report a case of FNH near the gallbladder in a 9-year-old girl. CEUS and CT showed that the lesion had a typical central stellate scar and spoke-wheel vessel structure. Based on imaging examination results and the clinical manifestation of premature breast development, we made a diagnosis of FNH. Histology revealed a disordered lobular structure with hyperplasia of fibrous tissue, confirming our diagnosis. To date, there are no clear guidelines for the treatment of FNH. In general, the stability of the lesion and its benignity can be evaluated by 6 months of follow-up (12). As the lesion in this case displayed evident enlargement within 6 months and the parents were concerned that it might continue to grow and cause symptoms, continued observation was not appropriate. Although surgical resection was feasible, the lesion was too close to the gallbladder to avoid cholecystectomy, and the parents hoped to preserve the gallbladder. There have been reports (8, 9) on the use of TAE for FNH in pediatric patients. However, TAE is associated with the risk of increased radiation exposure, and there are reports of residual (8, 13) lesions after TAE. To date, thermal ablation for FNH has only been reported in adults (14). Our team has also reported (15) the application of ultrasound-guided ablation for lesions near the gallbladder, thus avoiding the need for cholecystectomy. Accordingly, we chose to perform ultrasound-guided percutaneous MWA for this patient.

The difficulty of ablation lies in completely ablating lesions with an abundant blood supply. In this case, we chose MWA rather than RFA. First, we ablated the feeding artery, and successful ablation of the blood supply to the lesion was accurately evaluated by CEUS. After ablation of the feeding artery, we commenced ablation from the periphery to the interior of the lesion until complete ablation was achieved.

As the lesion was 1 mm away from the gallbladder, there was an additional challenge in this case of preventing thermal injury to the gallbladder. The lesion was near the bottom of the gallbladder, which meant that the gallbladder wall might be free from the liver. With the assistance of artificial ascites as well as an expansion tube and guide wire, we were able to push the gallbladder wall away to avoid injury during ablation. Thermal injury may also be assessed by CEUS to determine whether cholecystectomy should be applied. Overall, artificial ascites was useful for isolating the liver and the nearby intestine to avoid intestinal injury.

Color Doppler ultrasound and CEUS indicated abundant arterial blood supply to this lesion. Two strategies were used to avoid bleeding caused by biopsy and the possibility of implantation metastasis. First, biopsy was performed after ablation of the feeding vessels. Second, a coaxial needle was used. After the biopsy was completed, a microwave antenna was inserted through the coaxial needle shell to ablate the biopsy needle tract.

Although thermal ablation has been widely used for treating solid tumors such as hepatocellular carcinoma, there is only one report of FNH treated with radiofrequency ablation in adults and the procedure was only partially effective because a small residual was noted 2 months later (14). In our case, the 1-month MRI and 3-month ultrasound after ablation both showed no residual lesions, indicating that the procedure was effective.

In general, the findings from this case suggest that ultrasound-guided percutaneous MWA is a safe and effective treatment for FNH near the gallbladder in pediatric patients.

## Data Availability Statement

The original contributions presented in the study are included in the article/supplementary material, further inquiries can be directed to the corresponding author/s.

## Ethics Statement

Informed consent was obtained from the patient's parents prior to potential publication of this case report and any accompanying images.

## Author Contributions

Material preparation, data collection, and analysis were performed by ZY, QZ, SL, and SJ. The first draft of the manuscript was written by XY, DM, and KL. All authors commented on previous versions of the manuscript, read, approved the final manuscript, and contributed to the study conception and design.

## Conflict of Interest

The authors declare that the research was conducted in the absence of any commercial or financial relationships that could be construed as a potential conflict of interest.
